# Quantitative photoacoustic tomography

**DOI:** 10.1098/rsta.2009.0083

**Published:** 2009-08-13

**Authors:** Zhen Yuan, Huabei Jiang

**Affiliations:** Department of Biomedical Engineering, University of FloridaGainesville, FL 32611, USA

**Keywords:** photoacoustic tomography, reconstruction algorithm, laser, quantitative imaging, finite element, diffuse optical tomography

## Abstract

In this paper, several algorithms that allow for quantitative photoacoustic reconstruction of tissue optical, acoustic and physiological properties are described in a finite-element method based framework. These quantitative reconstruction algorithms are compared, and the merits and limitations associated with these methods are discussed. In addition, a multispectral approach is presented for concurrent reconstructions of multiple parameters including deoxyhaemoglobin, oxyhaemoglobin and water concentrations as well as acoustic speed. Simulation and *in vivo* experiments are used to demonstrate the effectiveness of the reconstruction algorithms presented.

## 1. Introduction

Biomedical photoacoustic tomography (PAT) is a potentially powerful modality that can offer high-resolution structural and functional imaging of tissue (Kruger & Liu 1994; Kruger *et al*. 1999; Oraevsky *et al*. 1999; Viator *et al*. 2002; Norton & Vo-Dinh 2003). In PAT, a short-pulse laser source is used to irradiate the tissue of interest. The laser-produced temperature rise and subsequent thermoelastic expansion of tissues generate an acoustic wave that is detected by ultrasound transducers along multiple boundary positions. A reconstruction algorithm is used to recover the photoacoustic (PA) images quantitatively or qualitatively. PAT has shown the potential to detect breast cancer, to probe brain functioning in small animals, and to assess vascular and skin diseases (Hoelen *et al*. 1998; Wang *et al*. 2002; Yin *et al*. 2004; Niederhauser *et al*. 2005; Manohar *et al*. 2007).

However, conventional PAT reconstruction methods are mostly qualitatively based, and can image only the distribution of absorbed light energy density, which is the product of both the local optical absorption coefficient and the optical fluence distribution within the irradiated medium. It is well known that it is the tissue absorption coefficient that directly correlates with tissue structural and functional information such as haemoglobin and blood oxygenation. Several recent studies have suggested that it is possible to recover the quantitative optical absorption coefficient map when conventional PAT is combined with a light transport model (Ripoll & Ntziachristos 2005; Cox *et al*. 2006; Yuan & Jiang 2006; Yin *et al*. 2007; Yuan *et al*. 2007). These studies have opened a new avenue to realize truly quantitative PAT by exploiting the spectral characteristics of specific chromophores in tissue, thus providing spatially resolved quantitative physiological and molecular information for valuable diagnostic decision making.

In addition to the optical properties and physiologically relevant tissue parameters, including deoxyhaemoglobin (Hb), oxyhaemoglobin (HbO_2_) and water (H_2_O), PAT can also capture tissue mechanical parameters such as acoustic velocity. Our recent studies in quantitative PAT have shown that the images of both acoustic velocity and absorbed light energy density can be recovered simultaneously (Jiang *et al*. 2006; Yuan *et al*. 2006; Yuan & Jiang 2007). This ability to recover both optical and acoustic properties not only provides more accurate reconstruction of optical properties than for qualitative PAT because of the elimination of the assumption of homogeneous acoustic velocity built into the qualitative PAT methods, but also adds the potential to better differentiate benign from malignant lesions as it is known that there exist significant differences in acoustic properties between normal and tumour tissues (Greenleaf & Bahn 1981).

In this paper, we will demonstrate and compare our quantitative PAT approaches that are based on the finite-element method. We will discuss the main characteristics of the reconstruction algorithms that allow for quantitative recovery of the optical absorption coefficient as well as the simultaneous reconstruction of the acoustic velocity and absorbed energy density.

In addition, advances in diffuse optical tomography (DOT) have shown that absorbers can be recovered with high accuracy when *a priori* spectral information provided by near-infrared spectroscopy is incorporated into the DOT reconstruction (Corlu *et al*. 2003, 2005; Li *et al*. 2004). Similarly, we are able to develop a new spectral approach that allows for direct simultaneous reconstruction of tissue chromophores and acoustic velocity using multiple-wavelength PA measurements. Owing to the use of multiple laser wavelengths, this approach provides more accurate parameter reconstruction, especially for acoustic velocity, as well as direct recovery of physiological parameters with enhanced accuracy over the Beer's law-based fitting methods (Laufer *et al*. 2007). We will demonstrate this multispectral PAT approach using simulations and *in vivo* experiments.

## 2. Overview of finite-element-based quantitative PAT reconstruction methods

### (a) Simultaneous recovery of acoustic velocity and absorbed energy density

Since our reconstruction algorithm for the simultaneous reconstruction of acoustic property and absorbed light energy density has been described in detail elsewhere (Jiang *et al*. 2006), we give only a brief outline here for context. The frequency-domain Helmholtz wave equation in an acoustically heterogeneous medium can be stated as

(2.1) $$\nabla^{2}p(r,\omega )+k_{0}^{2}(1+O)p(r,\omega )=\text{i}k_{0}\frac{v_{0}\beta \Psi (r)}{C_{p}}.$$

Here *p* is the acoustic pressure; *k*_0_=*ω*/*v*_0_ is the wavenumber, described by the angular frequency, *ω*, and the speed of the acoustic wave in a reference or coupling medium, *v*_0_; *β* is the thermal expansion coefficient; *C*_*p*_ is the specific heat; *Ψ* is the absorbed light energy density, which is the product of the optical absorption coefficient and incident optical fluence; and *O* is a coefficient that depends on both acoustic speed and attenuation as

(2.2) $$O=\frac{v_{0}^{2}}{v^{2}}-1+\frac{\text{i}}{k_{0}}\frac{\alpha v_{0}}{v^{2}},$$

in which *v* is the speed of the acoustic wave in the medium/tissue; and *α* is the acoustic attenuation coefficient. The finite-element discretization of equation (2.1) is stated as

(2.3) Ap=bΨ,

where the elements of the matrix ***A*** and ***b*** are expressed as

(2.4) $$\begin{array}{l} \begin{array}{l} A_{ij}=\langle \nabla \psi_{j}\cdot \nabla \psi_{i}\rangle -k_{0}^{2}\langle \psi_{j}\psi_{i}\rangle -k_{0}^{2}\langle \sum_{k}O_{\text{R},k}\psi_{k}\psi_{j}\psi_{i}\rangle \\ -\text{i}k_{0}^{2}\langle \sum_{l}O_{\text{I},l}\psi_{l}\psi_{j}\psi_{i}\rangle -\oint \left(\eta \psi_{j}+\gamma \frac{\partial^{2}\psi_{j}}{\partial \varphi^{2}}\right)\psi_{i}\;\text{d}s, \end{array}\\ B_{i}=-\text{i}k_{0}\frac{v_{0}\beta }{C_{p}}\langle \sum_{k}\Psi_{\text{R},k}\psi_{k}\psi_{i}\rangle +k_{0}\frac{v_{0}\beta }{C_{p}}\langle \sum_{l}\Psi_{\text{I},l}\psi_{l}\psi_{i}\rangle . \end{array}$$

Here *Ψ*, *O* and *p* are expressed by their real and imaginary components; *ψ*_*i*_ is the basis function; 〈 〉 indicates integration over the problem domain; and the second-order absorption boundary conditions are employed,

(2.5) $$\nabla \psi_{n}\cdot \hat{n}=\eta \psi_{n}+\gamma \frac{\partial^{2}\psi_{n}}{\partial^{2}\theta },$$

where $\eta =(-\text{i}k-3/2\rho +\text{i}3/8k\rho^{2})/(1-\text{i}/k\rho )$; $\gamma =(-\text{i}/2k\rho^{2})/(1-\text{i}/k\rho )$; and *θ* is the angular coordinate at radial position *ρ*. To obtain a matrix equation capable of inverse solution, a combined Marquardt and Tikhonov regularization scheme is used for the inverse calculation,

(2.6) $$(J^{\text{T}}J+\lambda^{\prime }I)\Delta Χ=J^{\text{T}}(p^{\text{o}}-p^{\text{c}}),$$

where $p^{\text{o}}={\left(p_{1}^{\text{o}},p_{2}^{\text{o}},\ldots ,p_{M}^{\text{o}}\right)}^{\text{T}}$; $p^{\text{c}}={\left(p_{1}^{\text{c}},p_{2}^{\text{c}},\ldots ,p_{M}^{\text{c}}\right)}^{\text{T}}$; $p_{i}^{\text{o}}$ and $p_{i}^{\text{c}}$ are observed and computed complex acoustic field data for *i*=1, 2, …, *M* boundary measurement locations; Δ*Χ* is the update vector for the optical and acoustic properties; ***J*** is the Jacobian matrix formed at the boundary measurement sites; *λ*′ is a scalar; and ***I*** is the identity matrix. Thus, here the image formation task is to update the absorbed light energy density and acoustic property distributions via iterative solution of equations (2.3) and (2.6), so that a weighted sum of the squared difference between the computed and measured acoustic pressure can be minimized.

For most cases where the heterogeneity/object has contrast in both the absorbed energy density and acoustic velocity, the energy density and acoustic velocity can be recovered at a single wavelength. This is very similar to what is seen in DOT, where both absorption and scattering coefficients can be reconstructed at a single wavelength when the objects have contrast in both the absorption and scattering. For such cases, crosstalk certainly exists, but it is not strong. This crosstalk is also dependent on the level of contrast for the two parameters. For the numerical and experimental cases we have previously investigated in PAT (Jiang *et al*. 2006; Yuan *et al*. 2006), the level of contrast used was relatively low, which explains the insignificant crosstalk that occurred in our reconstruction.

**Figure 1:**
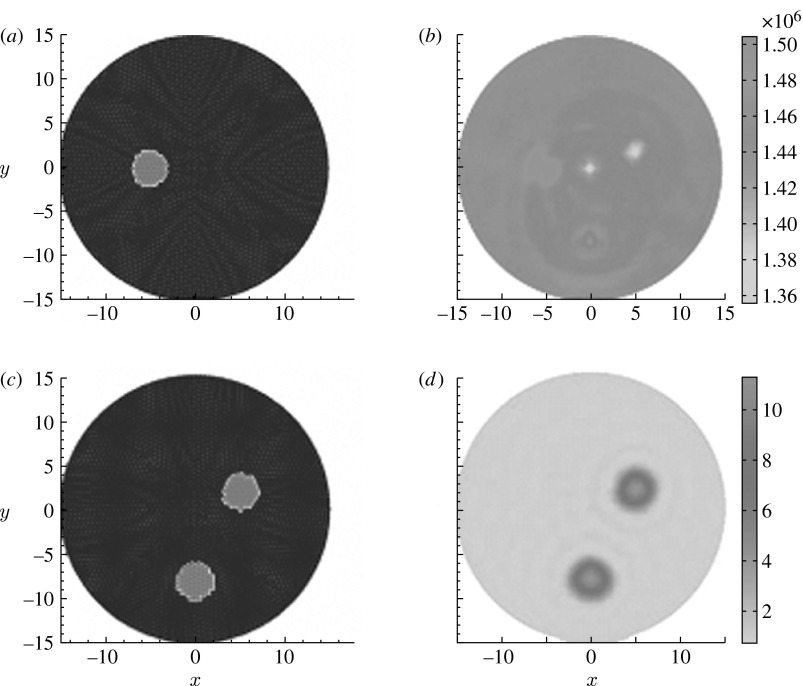
Reconstructed images using one wavelength measurement. (*a*,*c*) The true locations of the targets. The second column shows (*b*) recovered acoustic velocity (mm s^−1^) and (*d*) absorbed energy density (mJ mm^−3^) images, respectively.

However, for the cases where acoustic heterogeneity is quite different from the distribution of absorbed light energy density, our simulation tests indicate that the crosstalk cannot be minimized using one wavelength measurement. For our simulation tests, the circular background region (15 mm in radius) contains three circular targets (2 mm in radius each) positioned at 2 (top right), 9 (top left) and 6 o'clock (bottom). The top left inclusion has contrast for velocity, while the bottom and top right inclusions have contrast for absorbed energy density. The velocities for the targets and background are 1.54×10^6^ and 1.44×10^6^ mm s^−1^, respectively, while the absorbed energy densities for the background and targets are 1 and 10 mJ mm^−3^, respectively. We can see from the recovered images shown in figure 1 that the absorbed energy density distribution has some crosstalk effect on acoustic velocity, while the acoustic velocity distribution has no significant effect on the recovered absorbed energy density due to its low contrast.

### (b) Recovery of absolute absorption coefficient

We have developed two powerful algorithms (Yin *et al*. 2007; Yuan *et al*. 2007) that are able to recover the optical absorption coefficient from absorbed light energy density obtained by the conventional PAT method. These two algorithms are both based on the finite-element solution to the PA wave equation coupled with the photon diffusion equation.

#### (i) Algorithm 1

This algorithm includes two steps (Yin *et al*. 2007). The first is to obtain the map of absorbed light energy density through a model-based reconstruction algorithm that is based on the finite-element solution to the PA wave equation in the frequency domain. The second step is to obtain the distribution of optical fluence using a photon diffusion equation-based optimization procedure and to recover the distribution of the optical absorption coefficient from the optical fluence and the absorbed energy density obtained from the first step. In this method, the optical fluence is obtained using diffuse optical measurements along the boundary of the tissue/medium and through the iterative calculation of the following equations (Iftimia & Jiang 2000):

(2.7) $$\nabla \cdot D(r)\nabla \Phi (r)-\mu_{\text{a}}(r)\Phi (r)=-S(r),\;-D\nabla \Phi \cdot n=\alpha \Phi ,$$

(2.8) $${Χ}^{2}=\sum_{i=1}^{M}{\left(\Phi_{i}^{\left(\text{m}\right)}-\Phi_{i}^{\left(\text{c}\right)}\right)}^{2},$$

in which $\Phi_{i}^{\left(\text{m}\right)}$ and $\Phi_{i}^{\left(\text{c}\right)}$ are the measured and calculated optical fluence for *i*=1, 2, …, *M* boundary locations; *μ*_a_ is the optical absorption coefficient; and *D* is the optical diffusion coefficient. The goal of the second step is to obtain the distribution of the optical fluence within the entire imaging domain through an optimization procedure based on equations (2.7) and (2.8), which then allows the separation of the optical absorption coefficient from the optical fluence using the absorbed energy density obtained from the first step.

While algorithm 1 is very effective in recovering the absorption coefficient from the PAT data, there are some limitations associated with this approach. First, it requires additional diffuse light measurements, making the implementation of the hardware more complex. Second, the recovered results may depend on the accuracy of the distribution of the absolute absorbed energy density from conventional PAT.

#### (ii) Algorithm 2

To overcome these limitations mentioned above, a novel reconstruction approach that combines conventional PAT with the diffusion equation-based regularized Newton method for accurate recovery of optical properties has been proposed (Yuan *et al*. 2007). This approach is based on the following photon diffusion equation as well as the Robin boundary conditions, in terms of the absorbed energy density, *Ψ*=*μ*_a_*Φ*:

(2.9) ∇·D(r)∇(E(r)Ψ(r))−Ψ(r)=−S(r),

(2.10) −D∇(E(r)Ψ)·n=E(r)αΨ.

Here *E*(*r*)=1/*μ*_a_(*r*); *S*(*r*) is the incident point or distributed source term; and *D* is the optical diffusion coefficient. For the inverse computation, the so-called Tikhonov regularization sets up a weighted term as well as a penalty term in order to minimize the squared differences between computed and measured absorbed energy density values,

(2.11) $$\underset{Χ}{\text{min}}\{\|\Psi^{\text{c}}-\Psi^{\text{o}}{\|}^{2}+\beta \|L[E-E_{0}]{\|}^{2}\},$$

where ***L*** is the regularization matrix or filter matrix (Yuan *et al*. 2008); *β* is the regularization parameter; $\Psi^{\text{o}}={\left(\Psi_{1}^{\text{o}},\Psi_{2}^{\text{o}},\ldots ,\Psi_{N}^{\text{o}}\right)}^{\text{T}}$ and $\Psi^{\text{c}}={\left(\Psi_{1}^{\text{c}},\Psi_{2}^{\text{c}},\ldots ,\Psi_{N}^{\text{c}}\right)}^{\text{T}}$; $\Psi_{i}^{\text{o}}$ is the absorbed energy density obtained from PAT; and $\Psi_{i}^{\text{c}}$ is the absorbed energy density computed from equations (2.9) and (2.10) for *i*=1, 2, …, *N* locations within the entire PAT reconstruction domain. The initial estimate of the absorption coefficient can be updated based on the iterative Newton method as follows with *β*=1:

(2.12) $$▵(E)={\left(J^{\text{T}}J+\lambda^{\prime }I+L^{\text{T}}L\right)}^{-1}[J^{\text{T}}(\Psi^{\text{o}}-\Psi^{\text{c}})].$$

The basic idea of this approach is to incorporate the high-resolution PAT images into the DOT-like reconstruction, so that both the resolution and quantitative accuracy of optical image reconstruction are enhanced. However, the accuracy of the recovered absorption coefficient depends on the structural information obtained from PAT. Fortunately, our regularization-based diffuse optical reconstruction algorithm is immune to imperfect *a priori* spatial information (Yuan *et al*. 2008).

Further, it is noted that the scattering coefficient is assumed as constant/homogeneous for the use of both algorithms 1 and 2. While reconstructing only the absorption coefficient minimizes the non-uniqueness issue, it certainly imposes a limitation on our algorithms. This suggests that our methods may be applicable to cases where the scattering contrast is low or the objects having scattering contrast are small in size; otherwise, we may have to use DOT as we have indicated in our published work (Yin *et al*. 2007).

## 3. Development of spectrally resolved PAT

Early work in PAT focused on reconstructing tissue optical properties at several selected wavelengths (Laufer *et al*. 2007). A least-squares fitting algorithm was then used to estimate chromophore concentrations based on the recovered optical properties and Beer's law. However, our simulation tests indicate that the water content image cannot be effectively recovered using a fitting method. In our study, multispectral PAT is proposed to directly reconstruct chromophore concentrations in tissue without the use of a fitting method.

In multispectral PAT, the frequency-domain Helmholtz wave equation in an acoustically heterogeneous medium is written as

(3.1) $$\nabla^{2}p(r,\omega ,\lambda )+k_{0}^{2}(1+O)p(r,\omega ,\lambda )=\text{i}k_{0}\frac{v_{0}\beta \mu_{\text{a}}(r,\lambda )\Phi (r,\lambda )}{C_{p}},$$

where *λ* is the wavelength of the incident light. In addition, according to Beer's law, the wavelength-dependent tissue absorption can be expressed as

(3.2) $$\mu_{\text{a}}(\lambda )=\sum_{i=1}\epsilon_{i}(\lambda )c_{i},$$

where *c*_*i*_ is the concentration and *ϵ*_*i*_(*λ*) is the extinction coefficient of the *i*th chromophore (HbO_2_, Hb or H_2_O) at wavelength *λ*. In light of equation (3.2), equation (3.1) can be rewritten as

(3.3) $$\nabla^{2}p(r,\omega ,\lambda )+k_{0}^{2}(1+O)p(r,\omega ,\lambda )=\text{i}k_{0}\frac{v_{0}\beta \sum_{i=1}^{3}\epsilon_{i}(\lambda )c_{i}\Phi (r,\lambda )}{C_{p}}.$$

Thus, similar to equation (2.6), the inverse solution can be obtained by solving the equation

(3.4) $$(J^{\text{T}}J+\lambda^{\prime }I)\Delta Χ=J^{\text{T}}(p^{\text{o}}-p^{\text{c}}),$$

in which $\Delta Χ={\left(▵O_{1},▵O_{2},\ldots ,▵O_{n},▵c_{1,1},▵c_{1,2},\ldots ,▵c_{1,n},▵c_{2,1},▵c_{2,2},\ldots ,▵c_{2,n},▵c_{3,1},▵c_{3,2},\ldots ,▵c_{3,n}\right)}^{\text{T}}$ is the update vector for chromophores and acoustic velocity; *λ*′ is the regularization parameter determined by combined Marquardt and Tikhonov regularization schemes; and $p_{i}^{\text{o}}$ and $p_{i}^{\text{c}}$ are measured and calculated data for *i*=1, 2, …, *M* boundary locations. For each acoustic frequency *ω* within each incident optical wavelength *λ*, these are written as

(3.5) $$p^{\text{o}}={\left(p_{1}^{\text{o}},p_{2}^{\text{o}},\ldots ,p_{M}^{\text{o}}\right)}^{\text{T}}|(\omega ,\lambda ),\;\;p^{\text{c}}={\left(p_{1}^{\text{c}},p_{2}^{\text{c}},\ldots ,p_{M}^{\text{c}}\right)}^{\text{T}}|(\omega ,\lambda ).$$

The Jacobian matrix, ***J***, is denoted as $J=[{\tilde{J}}_{O,\lambda ,\omega },{\tilde{J}}_{c_{i},\lambda ,\omega }]$, where ${\tilde{J}}_{O,\lambda ,\omega }$ and ${\tilde{J}}_{c_{i},\lambda ,\omega }$ represent the Jacobian submatrix for acoustic velocity and different chromophores, respectively. It should be noted that, in this reconstruction algorithm, we assume that the optical fluence *Φ* can be estimated through a photon diffusion equation during the image reconstruction procedure (Yuan & Jiang 2006).

## 4. Results and discussion

In this section, we show reconstruction results that demonstrate the feasibility of the multispectral PAT described above. The multispectral PAT approach is evaluated using several simulations and small animal experiments.

**Figure 2:**
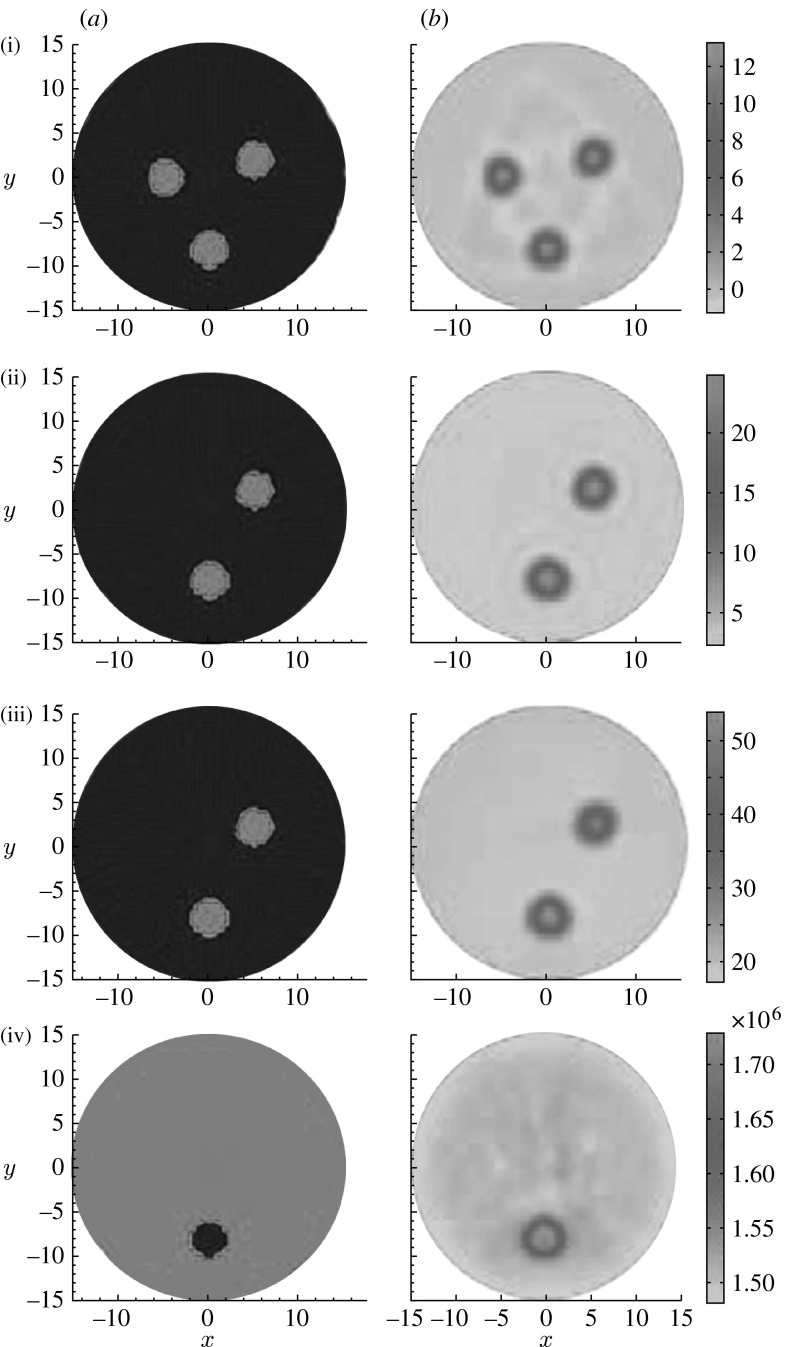
(*a*) Exact and (*b*) reconstructed images using six optical wavelengths. The first to the fourth rows show (i) Hb (μM), (ii) HbO_2_ (μM), (iii) H_2_O (%) and (iv) acoustic velocity (mm s^−1^) images, respectively.

For the simulations, we use a circular background (15 mm in radius) containing three circular targets (2 mm in radius each), where each inclusion had different contrast for each given parameter, namely Hb, HbO_2_, H_2_O and acoustic velocity. The chromophore concentrations and velocity used for all the test cases are listed in table 1. Six optical wavelengths (633, 682, 723, 850, 854 and 930 nm) were chosen for the spectrally resolved PAT. The ‘measured’ data were generated using a forward computation with 2 per cent random Gaussian noise. A total of 120 receivers were equally distributed along the boundary of the circular background. Fifty frequencies ranging from 50 to 540 kHz were used at each optical wavelength for each receiver. The extinction coefficient for each chromophore used was taken from S. Pahl (2003, http://omlc.ogi.edu/spectra/index.html). The images in figure 2*a*(i)–(iv) show the true locations of the targets including Hb, HbO_2_, H_2_O and acoustic velocity. The reconstructed images shown in figure 2*b*(i)–(iv) correspond to Hb, HbO_2_, H_2_O and acoustic velocity. We see from figure 2 that the crosstalk errors between the chromophore concentrations and acoustic velocity have been effectively reduced using our quantitative PAT approach.

**Figure 3:**
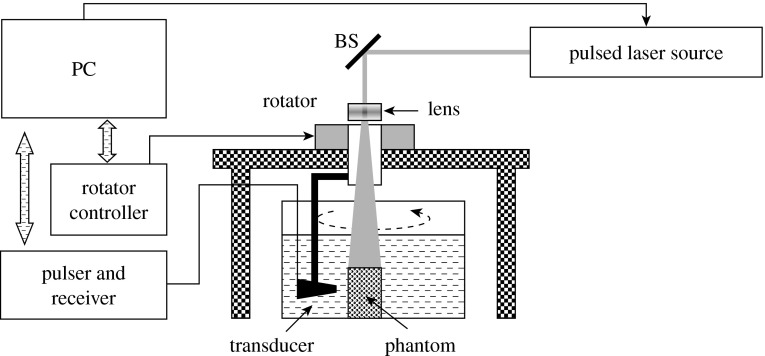
Schematic of our PAT system. BS, beam splitter; PC, personal computer.

Animal experiments were performed using a PAT system that has been described in detail elsewhere (Yuan & Jiang 2006). In this system (figure 3), pulsed light from a Ti:sapphire laser (Symphotic Tii Corp., Camarillo, CA) is used to irradiate the tissue and generates an acoustic pressure wave. A 1 MHz transducer (GE Panametrics, Waltham, MA) is used to receive the acoustic signals. The transducer and the animal (oxygen provided) are immersed in a water tank. A rotary stage rotates the receiver relative to the centre of the tank. One set of data is taken at 120/360 positions when the receiver is scanned circularly over 360°. The complex wavefield signal is first amplified by a preamplifier, and then amplified further by a pulser/receiver. A data acquisition board converts it into a digital signal, which is fed to a computer. The radius of the receiver motion path was 100 mm in the animal experiments conducted. The entire data acquisition is realized through C programming. In the current version of the system, data collection for a total of 120 measurements requires approximately 2 min.

**Figure 4:**
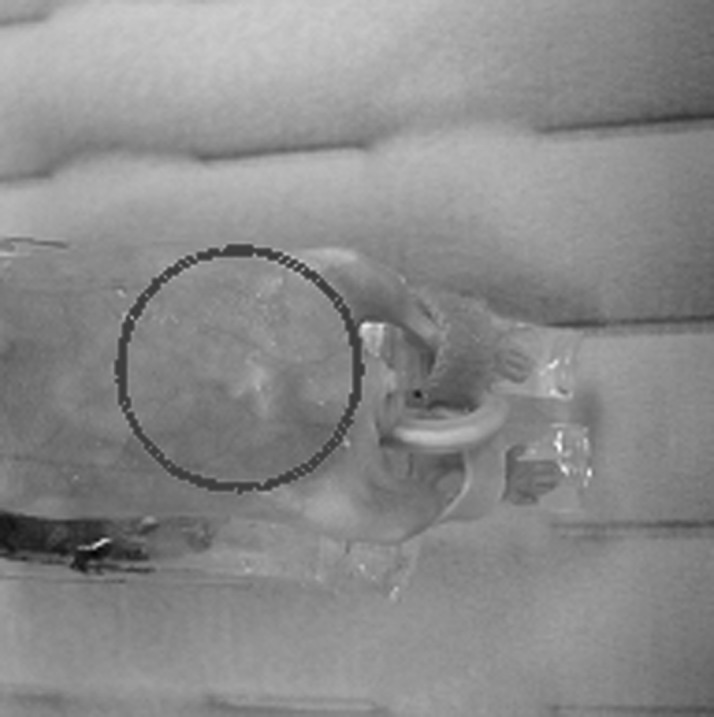
Photograph showing the imaging region for a mouse with an implanted subcutaneous tumour.

**Figure 5:**
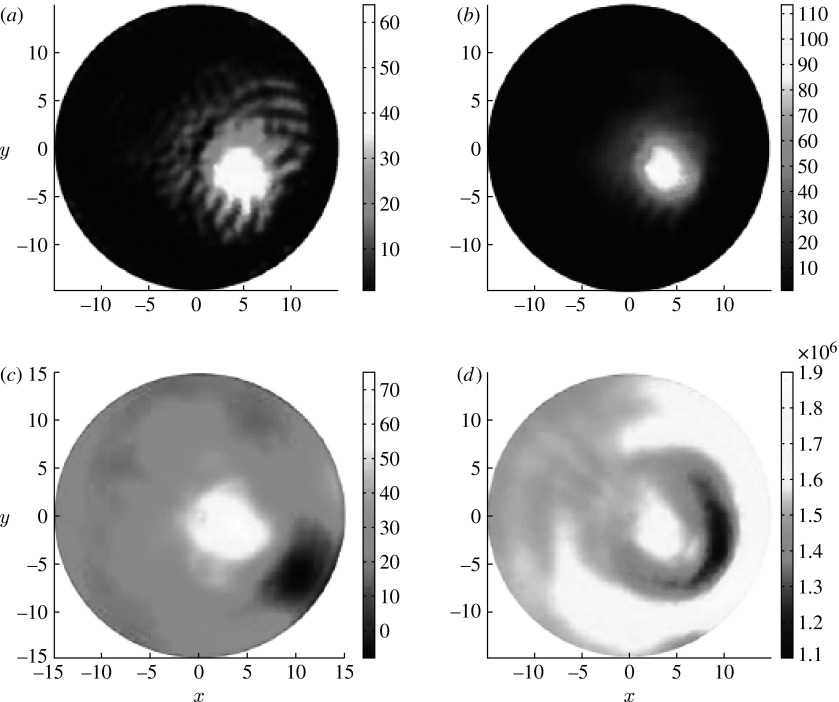
Reconstructed *in vivo* images of (*a*) Hb (μM), (*b*) HbO_2_ (μM), (*c*) H_2_O (%) and (*d*) acoustic velocity (mm s^−1^) from a mouse with a subcutaneous tumour.

Figure 4 shows a photograph of a typical mouse with an implanted subcutaneous tumour. Five optical wavelengths (755, 800, 860, 900 and 930 nm) were used for the measurements. Figure 5*a–d* presents the reconstructed *in vivo* Hb, HbO_2_, H_2_O and acoustic velocity images. We see that the tumour is remarkably imaged with the highest contrast in Hb and HbO_2_. In addition, the H_2_O image is also effectively recovered with better quality compared with that from diffuse optical imaging (Corlu *et al*. 2005). The contrast in velocity between the tumour and surroundings (figure 5*d*) is apparently lower than that for the physiological parameter images (figure 5*a–c*). The *in vivo* results shown here further validate the merits of the multispectral PAT method.

For all the tests, multispectral PAT can effectively minimize the crosstalk and improve the image quality. The direct reconstruction method has demonstrated that improved quantification of chromophore concentrations is achieved with multi-wavelength measurements when spectral priors are incorporated into the inverse reconstruction.

## 5. Conclusions

In this paper, we have described and compared several algorithms for realizing quantitative PAT. In addition, we have demonstrated experimental evidence that both physiological and acoustic property images can be obtained using our multispectral PAT approaches. The multispectral PAT approach presented provides an efficient means of concurrent reconstruction of multiple parameters including different chromophore concentrations and acoustic velocity. Images generated from both noisy simulated data and measured *in vivo* data have been provided. It is clear from the reconstructed results that the crosstalk issue for recovery of multiple parameters can be reduced or minimized using multispectral PAT approaches. We also found that the selection of the number of wavelengths (Corlu *et al*. 2003, 2005) as well as the wavelength range is critical in obtaining reliable and accurate reconstruction of multiple parameters, and the findings will be discussed in detail elsewhere (Yuan & Jiang 2009). Nonetheless, the ability to reconstruct multiple-parameter images may provide us with a convenient tool to quantify physiological function, disease progression or response to intervention.

## Figures and Tables

**Table 1:** Chromophore concentrations and acoustic velocity for test objects.

object position	HbO_2_ (μM)	Hb (μM)	H_2_O (%)	acoustic velocity (×10^6^ mm s^−1^)
background	6	2	20	1.44
top right	20	12	50	1.44
top left	6	12	20	1.44
bottom middle	20	12	50	1.74

## References

[bib1] Corlu A., Durduran T., Choe R., Schweiger M., Hillman E., Arridge S.R., Yodh A.G. (2003). Uniqueness and wavelength optimization in continuous-wave multispectral diffuse optical tomography. Opt. Lett.

[bib2] Corlu A., Choe R., Durduran T., Lee K., Schweiger M., Arridge S.R., Hillman E., Yodh A.G. (2005). Diffuse optical tomography with spectral constraints and wavelength optimization. Appl. Opt.

[bib3] Cox B., Arridge S., Kostli K., Beard P. (2006). 2D quantitative photoacoustic image reconstruction of absorption distribution in scattering media. Appl. Opt.

[bib19] Greenleaf J.F., Bahn R.C. (1981). Clinical imaging with transmissive ultrasound computerized tomography. IEEE Trans. Biomed. Eng.

[bib4] Hoelen C.G.A., de Mul F.F., Pongers R., Dekker A. (1998). Three-dimensional photoacoustic imaging of blood vessels in tissues. Opt. Lett.

[bib5] Iftimia N., Jiang H. (2000). Quantitative optical image reconstruction of turbid media by use of direct-current measurements. Appl. Opt.

[bib6] Jiang H., Yuan Z., Gu X. (2006). Spatially varying optical and acoustic property reconstruction using finite element-based photoacoustic tomography. J. Opt. Soc. Am. A.

[bib7] Kruger R.A., Liu P. (1994). Photoacoustic ultrasound: pulse production and detection in 0.5% Liposyn. Med. Phys.

[bib8] Kruger R.A., Reinecke D., Kruger G. (1999). Thermoacoustic computed tomography—technical considerations. Med. Phys.

[bib9] Laufer J., Delpy D., Elwell C., Beard P. (2007). Quantitative spatially resolved measurement of tissue chromophore concentrations using photoacoustic spectroscopy: application to the measurement of blood oxygenation and haemoglobin concentration. Phys. Med. Biol.

[bib10] Li A., Zhang Q., Culver J.P., Miller E.L., Boas D.A. (2004). Reconstruction chromosphere concentration images directly by continuous-wave diffuse optical tomography. Opt. Lett.

[bib11] Manohar S., Vaartjes S.E., van Hespen J.C.G., Klaase J.M., van den Engh F.M., Steenbergen W., van Leeuwen T.G. (2007). Initial results of *in vivo* non-invasive cancer imaging in the human breast using near-infrared photoacoustics. Opt. Express.

[bib12] Niederhauser J.J., Jaeger M., Lemor R., Weber P., Frenz M. (2005). Combined ultrasound and optoacoustic system for real-time high-contrast vascular imaging *in vivo*. IEEE Trans. Med. Imaging.

[bib13] Norton S.J., Vo-Dinh T. (2003). Optoacoustic diffraction tomography: analysis of algorithms. J. Opt. Soc. Am. A.

[bib14] Oraevsky A., Andreev V., Karabutov A., Fleming D., Gatalica Z., Singh H., Esenaliev R. (1999). Laser optoacoustic imaging of the breast: detection of cancer angiogensis. Proc. SPIE.

[bib16] Ripoll J., Ntziachristos V. (2005). Quantitative point source photoacoustic inversion formulas for scattering and absorbing medium. Phys. Rev. E.

[bib17] Viator J.A., Au G., Paltauf G., Jacques S., Prahl S., Ren H., Chen Z., Nelson J. (2002). Clinical testing of a photoacoustic probe for port wine stain depth determination. Lasers Surg. Med.

[bib18] Wang X., Xu Y., Xu M., Yokoo S., Fry E., Wang L. (2002). Photoacoustic tomography of biological tissues with high cross-section resolution: reconstruction and experiment. Med. Phys.

[bib20] Yin B., Xing D., Wang Y., Zeng Y., Tan Y., Chen Q. (2004). Fast photoacoustic imaging system based on 320-element linear transducer array. Phys. Med. Biol.

[bib21] Yin L., Wang Q., Zhang Q., Jiang H. (2007). Tomographic imaging of absolute optical absorption coefficient in turbid medium using combing photoacoustic and diffusing light measurements. Opt. Lett.

[bib22] Yuan Z., Jiang H. (2006). Quantitative photoacoustic tomography: recovery of optical absorption coefficient map of heterogeneous medium. Appl. Phys. Lett.

[bib25] Yuan Z., Jiang H. (2007). Three-dimensional finite element-based photoacoustic tomography: reconstruction algorithm and simulations. Med. Phys.

[bib26] Yuan Z., Jiang H. (2009). Simultaneous recovery of tissue physiological and acoustic properties and the criteria for wavelength selection in multispectral photoacoustic tomography. Opt. Lett.

[bib23] Yuan Z., Zhang Q., Jiang H. (2006). Simultaneous reconstruction of acoustic and optical properties of heterogeneous media by quantitative photoacoustic tomography. Opt. Express.

[bib24] Yuan Z., Wang Q., Jiang H. (2007). Reconstruction of optical absorption coefficient maps of heterogeneous media by photoacoustic tomography coupled with diffusion equation based regularized Newton method. Opt. Express.

[bib27] Yuan Z., Zhang Q., Sobel E., Jiang H.B. (2008). Tomographic x-ray-guided three-dimensional diffuse optical tomography of osteoarthritis in the finger joints. J. Biomed. Opt.

